# Incorporation of FGF-2 into Pharmaceutical Grade Fucoidan/Chitosan Polyelectrolyte Multilayers

**DOI:** 10.3390/md18110531

**Published:** 2020-10-26

**Authors:** Natalie L. Benbow, Samuel Karpiniec, Marta Krasowska, David A. Beattie

**Affiliations:** 1 Future Industries Institute, University of South Australia, Mawson Lakes, SA 5095, Australia; 2 Marinova, Cambridge, TAS 7170, Australia

**Keywords:** fucoidan, chitosan, fibroblast growth factor-2, polyelectrolyte multilayer, infrared spectroscopy, quartz crystal microbalance

## Abstract

Biopolymer polyelectrolyte multilayers are a commonly studied soft matter system for wound healing applications due to the biocompatibility and beneficial properties of naturally occurring polyelectrolytes. In this work, a popular biopolymer, chitosan, was combined with the lesser known polysaccharide, fucoidan, to create a multilayer film capable of sequestering growth factor for later release. Fucoidan has been shown to act as a heparin-mimic due to similarities in the structure of the two molecules, however, the binding of fibroblast growth factor-2 to fucoidan has not been demonstrated in a multilayer system. This study assesses the ability of fucoidan to bind fibroblast growth factor-2 within a fucoidan/chitosan polyelectrolyte multilayer structure using attenuated total internal reflectance infrared spectroscopy and quartz crystal microbalance with dissipation monitoring. The fibroblast growth factor-2 was sequestered into the polyelectrolyte multilayer as a cationic layer in the uppermost layers of the film structure. In addition, the diffusion of fibroblast growth factor-2 into the multilayer has been assessed.

## 1. Introduction

The use of growth factors in tissue engineering has been widely studied due to their ability to encourage healing and tissue growth [1]. A key example of an application where growth factors could provide significant benefit is for enhancing healing of chronic wounds. Chronic wounds are a significant issue in healthcare, severely affecting quality of life of affected people and contributing 2% to the total health care expenditure in countries such as Australia, the U.K. and the U.S.A. [2,3]. Applying growth factors to a wound site could promote healing by mimicking a healthy body’s natural response to injury, i.e., by delivering the growth factors to a wound bed, the migration and proliferation of cells will be promoted. Of particular interest is fibroblast growth factor-2 (FGF-2) which is one of several biomolecules that are responsible for signalling cell migration and proliferation in the body [4]. FGF-2 is part of the 22-member FGF-family and has been shown to promote angiogenesis, cell proliferation, migration, and differentiation [5]. FGF-2 is commonly studied as a model growth factor in materials science studies for wound healing applications [6,7,8,9,10] and has been shown to reduce healing time [11,12]. However, there are limitations in regards to the delivery of growth factors, i.e., the growth factor must be protected from degradation and delivered at the optimal stage of healing at the right dose and for the correct duration [13].

Polyelectrolyte multilayers (PEMs) are surface coatings that offer potential advantages for delivering growth factors in many biomedical applications, such as vascular repair [14] and also in wound healing. When deployed for wound treatment, the architecture of the PEM film can be designed to give a burst release [15,16,17] or slow diffusion of the peptides from the film [18]. Delivery of target molecules can be partially controlled through changing the conditions of multilayer assembly including ionic strength [19], pH [16,20,21], temperature [22] and polyelectrolyte species [8]. In addition, PEMs can be applied to many surfaces including flat planes [23], nanoparticles [24] and nanocapsules [25,26], and three-dimensional (3D) porous scaffolds [27]. Furthermore, growth factors often show no conformational change or reorganisation upon binding to polyelectrolytes in PEMs when absorbed from solution at room temperature [5,28,29].

The goal of our study into growth factor-loaded PEM films was two-fold. First, to create a multilayer that will act as a natural matrix for the growth factor. Second, to create a multilayer host film that could have potential for synergistic therapeutic effect. Both of these goals were addressed through the choice of the polymers used to create the multilayers. Our work has made use of two naturally occurring biopolymers as the two major multilayer components; fucoidan and chitosan. The latter of these two polymers finds widespread application and study in the area of would healing, due to its properties: non-toxic, biocompatible, biodegradable, and anti-fungal. Past studies have also shown that chitosan promotes fibroblast proliferation [30,31]

Fibroblast growth factor-2 (FGF-2) needs to be embedded within the PEM to ensure it is protected from degradation but can remain biologically active [32]. The heparin binding site of FGF-2 is a highly positive environment due to the basic amino acid groups present [28] and can interact with negatively charged sulfate groups in heparin and heparin-mimics, including fucoidan [33,34,35,36,37,38] to create a ternary complex with the growth factor cell receptors. This complex is required for FGF-2 to be bioactive [33] and has been shown to improve skin healing [39]. Combining fucoidan and growth factors has been attempted by other groups, and has been shown to improve angiogenesis [40,41] and cell proliferation [42]. A single previous example exists of fucoidan combined with a growth factor (vascular endothelial growth factor) in a multilayer film [43]. This study found improved anti-thrombic properties and re-endothelialisation of a decellurised heart valve in response to interaction with this multilayer system.

Furthermore, fucoidan can be pro-angiogenic [38], anti-inflammatory, anti-viral, and promotes anti-bacterial activity of other molecules [44,45]. In addition, it can be pro- or anti-coagulant depending on molecular weight [46], promotes cell proliferation and migration [35,39,47] and can be immuno-modulating depending on the molecular weight and structure [48,49,50]. Fucoidan also inhibits MMP-2 (matrix metalloproteinase-2), an enzyme that degrades type IV collagen, a major component of basement membranes upon which the epithelium is constructed [51]. Meanwhile, chitosan has been shown to offer protection of FGF-2 against denaturation from heat, proteolysis and acid [52]. Chitosan has been shown to accelerate healing in diabetic mice (chronic wound models), acting as a delivery method for FGF-2 to further promote healing [52,53].

We have used attenuated total internal reflectance Fourier transform infrared spectroscopy and quartz crystal microbalance with dissipation monitoring to investigate the fucoidan/chitosan polyelectrolyte multilayers as a potential reservoir for FGF-2. Additionally, the permeation of FGF-2 into the multilayer was compared to the permeation of lysozyme. Lysozyme is a small protein of similar size and charge to FGF-2 and has been shown to permeate into these multilayers in our previous work [54]. Our overall aim has been to determine how FGF-2 can be incorporated into multilayer films, either pre-prepared for deployment in biomaterials applications, or as a sink within a biofluid environment to harvest and protect released FGF-2, to allow it to survive for longer in the environment in which it needs to act.

## 2. Results

### 2.1. QCM-D

**Figure 1:**
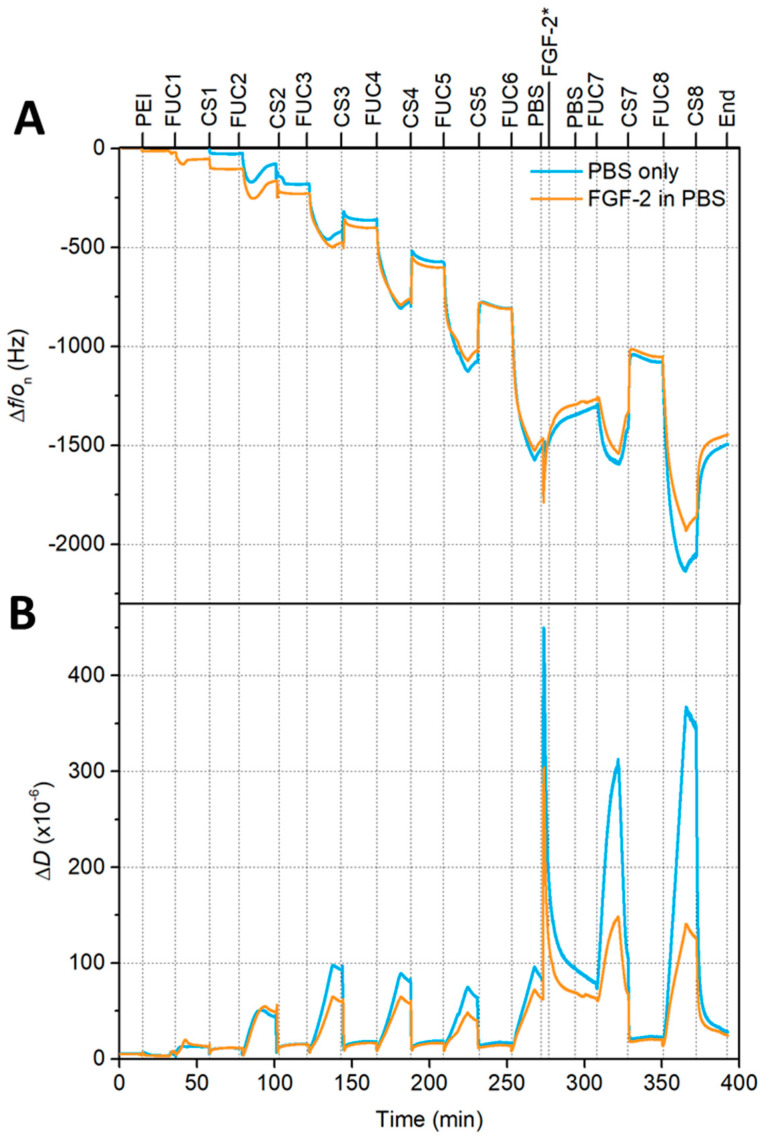
A representative QCM-D (Quartz crystal microbalance with dissipation) plot of frequency (**A**) and dissipation (**B**) for build-up of 8 bilayers for FUC/CS (fucoidan/chitosan) multilayers on gold sensors (5th overtone) where the PEM (polyelectrolyte multilayer) exposed to FGF-2 (fibroblast growth factor-2) are indicated by an orange line, whilst the control PEM exposed to only PBS (phosphate buffer solution) is marked with a blue line. The vertical dashed lines indicate the start of the adsorption step of each polymer. * Indicates were FGF-2 adsorption began for the data set presented in orange.

**Figure 2:**
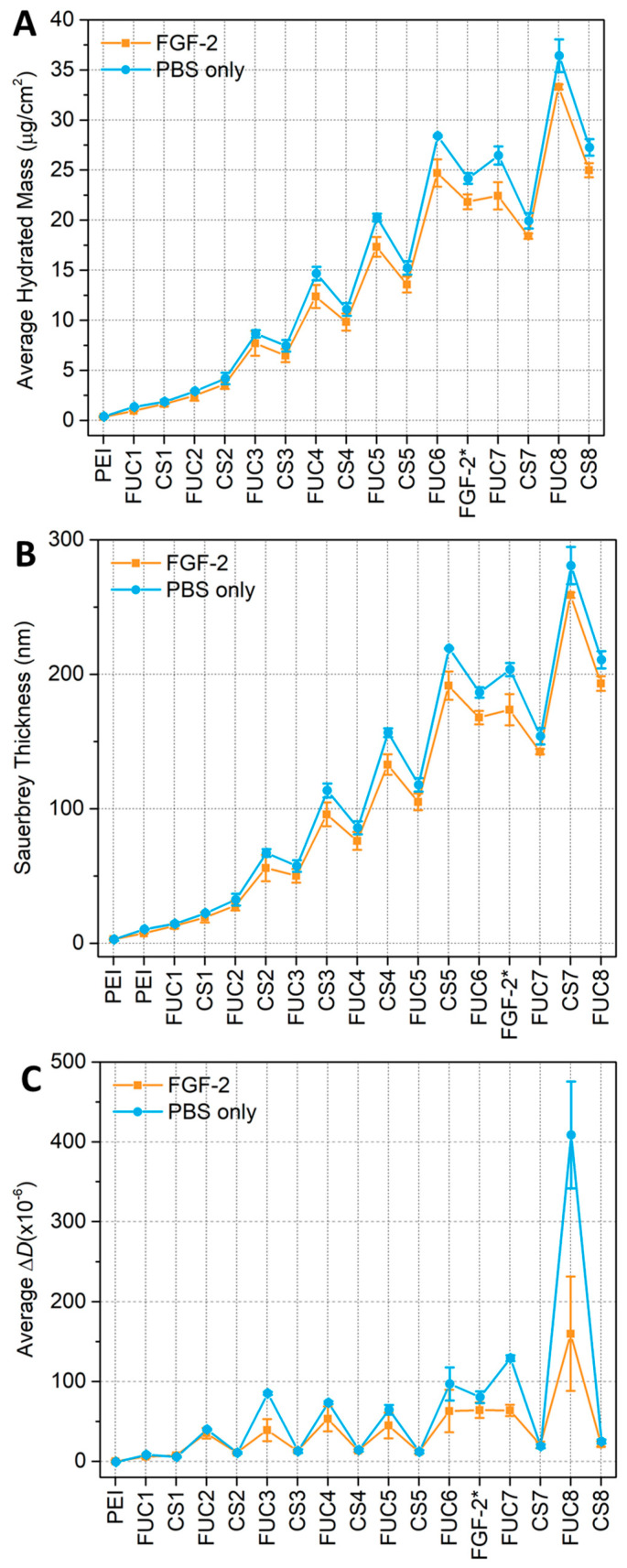
QCM-D calculations for (**A**) the average hydrated Sauerbrey mass, (**B**) the average Sauerbrey thickness; and (**C**) the average dissipation of the 8 bilayer FUC/CS PEM with embedded FGF-2 (orange with square markers–and highlighted with an asterisk on the x-axis) and PBS without FGF-2 (blue with circle markers).

Gorouhi et al. found that epidermal growth factor was still effective at promoting cell proliferation when covered by two bilayers in a PEM [32], thus, FGF-2 was placed at bilayer 6 of 8 in our study. Quartz crystal microbalance with dissipation (QCM-D) monitoring experiments were performed to monitor the build-up of the multilayers, either with, or without, the inclusion of the growth factor. In the case of a growth factor-embedded film, the PEM was constructed with sequential exposure of the substrate to the two polysaccharides until bilayer 5.5 (fucoidan terminating). The PEM was then exposed to PBS (phosphate buffer solution) for 5 min followed by 15 min FGF-2 25 μg∙mL^−1^ solution, then a 5 min rinse with PBS to remove any unbound FGF-2. This PBS/FGF-2/PBS cycle substituted as the 6th CS (chitosan) layer. The PEM construction was continued as normal from the fucoidan terminating seventh bilayer, until 8 bilayers were deposited. For the non-embedded/blank system (i.e., without growth factor incorporation), the multilayer was exposed to PBS at the 5.5. bilayer formation point, and then chitosan was added in place of the growth factor, prior to continuing the formation until 8 bilayers were formed. Two independent experiments were performed with a total of two sensors for each condition. A representative measurement is shown in Figure 1, whilst the average Sauerbrey thickness, average hydrated mass and average dissipation can be found in Figure 2.

The QCM-D data presented in Figure 1 indicate that the fucoidan layers show an increase in both frequency magnitude and dissipation, which becomes larger with increasing layer number, reflecting the supra-linear growth of the multilayer. The chitosan layers show a sharp decrease in both frequency magnitude and dissipation. In both panels of Figure 1 a sharp spike can be seen when the multilayer is initially exposed to PBS. The frequency magnitude and dissipation then decrease to values less than the 6 bilayer fucoidan-terminating PEM within 5 min for both multilayers exposed to FGF-2 in PBS and PBS only. There is a small but continual decrease in the frequency when the film is exposed to PBS (both with FGF-2 and without) indicating a mass loss during this time. However, QCM-D cannot distinguish between polymer mass and water mass loss. Our previous work has shown that 10 bilayer fucoidan/chitosan multilayers experience both a degree of mass loss and swelling when exposed to PBS [54]. However, any swelling occurring here (Figure 1) cannot be seen in the frequency measurements after the initial spike, in fact, a decrease in thickness is observed (see Figure 2).

The pattern seen in the early layers, of lower frequency and dissipation for chitosan layers, and a sharp increase in both frequency magnitude and dissipation for fucoidan layers, continues after PBS exposure with some differences, the first fucoidan layer after PBS exposure shows the same frequency as the previous fucoidan layer. The dissipation increases sharply with the fucoidan adsorption after PBS. The seventh and eighth chitosan layers show a decrease from the previous reported fucoidan layers in the frequency magnitude and dissipation values. The hydrated mass calculated using the Sauerbrey relation and the Sauerbrey thickness (panel A and B of Figure 2), show that the incorporation of FGF-2 does not have a significant impact on these two attributes of the multilayer compared to a PEM only exposed to PBS. The hydrated mass can be seen to follow a linear profile up to bilayer 3 where a saw-tooth profile emerges, with the chitosan layers having a lower hydrated mass than the previous fucoidan layers. Upon exposure to PBS the hydrated mass can be seen to decrease, following this exposure the continued build-up of the multilayer again shows a saw-tooth profile similar to before the PBS/FGF-2/PBS cycle. The PEM that was only exposed to PBS appears to show a marginally higher adsorbed mass, however, this was present prior to PBS exposure so is likely caused by a variation in the samples themselves.

Similarly, the thickness data in panel B of Figure 2, show a similar profile to the mass calculations prior to PBS exposure, where the linear profile ends at bilayer 3 and the saw-tooth begins. Again, when chitosan is adsorbed to the fucoidan-terminating multilayer the film becomes thinner, when the next layer of fucoidan is adsorbed the entire multilayer become thicker. When exposed to PBS, the thickness of the multilayer increases, upon the adsorption of the first subsequent bilayer pair, consisting of fucoidan then chitosan, the thickness decreases. These changes in thickness and mass show a swelling and deswelling profile. No polymer is added during the PBS only rinse and very little mass is added during the PBS/FGF-2/PBS cycle, yet the mass and thickness both increase.

The average dissipation data is presented in panel C of Figure 2, the PEMs with and without growth factor have very similar dissipations after being exposed to FGF-2 in PBS or only PBS. Though, this small difference may be due to variation in the samples that exists prior to the PBS exposure. For the seventh layer of fucoidan, there was a significant difference in the dissipation with the PBS only system having a much greater dissipation than the multilayer with embedded FGF-2, the eighth fucoidan layer shows a similar difference. However, the dissipation of the seventh and eighth chitosan layers are the same for both the multilayer with embedded FGF-2 and the multilayer that was only exposed to PBS.

### 2.2. ATR FTIR

**Figure 3:**
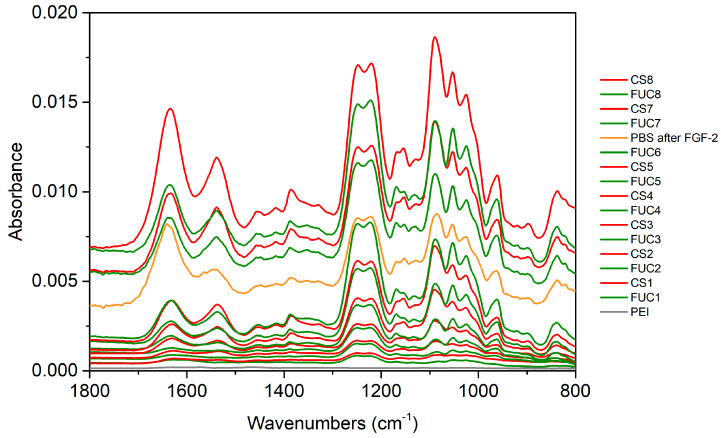
ATR FTIR (attenuated total reflectance Fourier transform infrared) spectra of build-up of a 8 bilayer FUC/CS PEM on a ZnSe IRE, where FGF-2 was embedded at bilayer 6. The grey line represents the spectrum of PEI, green lines represent FUC layers, red lines represent CS, and the orange line shows the spectrum of FGF-2 after a 5 min PBS rinse.

The ATR FTIR (attenuated total reflectance Fourier transform infrared) spectroscopy build-up and growth factor embedding experiments were performed with two independent repeats on a ZnSe IRE (internal reflection element). The spectra in Figure 3 show the PEM build-up proceeded as expected up to 5.5 bilayers (some of the later layer spectra have been offset vertically for clarity). The spectra show the characteristic peaks of fucoidan and chitosan (assigned previously [55,56,57]). The characteristic peaks assigned to chitosan are the amide I/C = O at 1633 cm^−1^ and amide II at 1535 cm^−1^. While the characteristic peaks attributed to the sulfate stretching vibration are at 1249 cm^−1^ and 1220 cm^−1^. Other peaks of interest include the overlapping peaks at 1167 cm^−1^ assigned to C–O–C stretching vibration fucoidan and 1152 cm^−1^ assigned to C–O–C/C–N stretching vibrations of chitosan. The glycosidic linkages and skeletal C–O stretching vibrations is encompassed by the peaks at 1090 cm^−1^, 1051 cm^−1^ and 1025 cm^−1^ for both polysaccharides. A complete list of peak positions and assignments can be found in Table 1.

During the individual layer adsorption steps, when fucoidan was adsorbed an increase in the sulfonate stretching band at 1238 cm^−1^ is clearly seen along with increases in the lower wavenumbers of the glycosidic linkage region (1100–950 cm^−1^). As chitosan adsorbs the greatest differences are increases in the entire glycosidic linkage region and in the amide I and II bands. These amide bands decrease slightly upon subsequent fucoidan adsorption. This decrease is the result of stripping of chitosan from the multilayer upon adsorption of fucoidan. Stripping of polyelectrolytes has been observed in fucoidan/chitosan multilayers in past work from this group (and is commonly observed more broadly with polyions of dissimilar molecular weights), when fucoidan of much lower molecular weight has been used (see [56] and references contained therein).

The spectrum of the PBS rinse after FGF-2 adsorption displays some significant changes. At 5.5 bilayers the PEM has been calculated to be 192 ± 10 nm thick using the Sauerbrey equation from the QCM-D measurements presented in Figure 2, panel B. When the film was exposed to PBS/FGF-2/PBS the Sauerbrey thickness decreased to 168 ± 5 nm, despite an initial spike caused by PBS. The significant decrease in absorbance after the PBS/FGF-2/PBS cycle indicates mass loss of polysaccharides from the film. The peak heights of the sulfonate bands and the glycosidic region match that of the 5th bilayer, chitosan terminating film suggesting much of the previously adsorbed fucoidan layer has been removed. In addition, increases in the amide I/II bands indicating that FGF-2 adsorbed to the multilayer.

Following the PBS/FGF-2/PBS cycle the multilayer build-up was continued. The first fucoidan layer after this cycle has the same peak heights as the preceding fucoidan layer that was diminished by the adsorption of the FGF-2 layer (and associated PBS rinse cycles). The characteristic peaks in the next chitosan layer spectra increase very little, whilst the next bilayer appears to return to a more typical build-up as seen with the early layers prior to PBS/FGF-2 exposure. There is one additional difference in the final chitosan layer; the sulfate band attributed to fucoidan increases, likely due to underlying chitosan peaks in the spectrum and the large amount of chitosan that appears to be adsorbing to this layer. It is unlikely to be a result of the penetration depth of the evanescent wave as the *d*_p_ of the ZnSe IRE is approximately 850 nm (*ñ* = 1650 cm^−1^) and the Sauerbrey thickness of the multilayer at the time of formation of the eighth chitosan layer is 193 ± 5 nm for the film with embedded FGF-2 (see the supporting information of our previous work [54] for calculations of *d*_p_).

**Figure 4:**
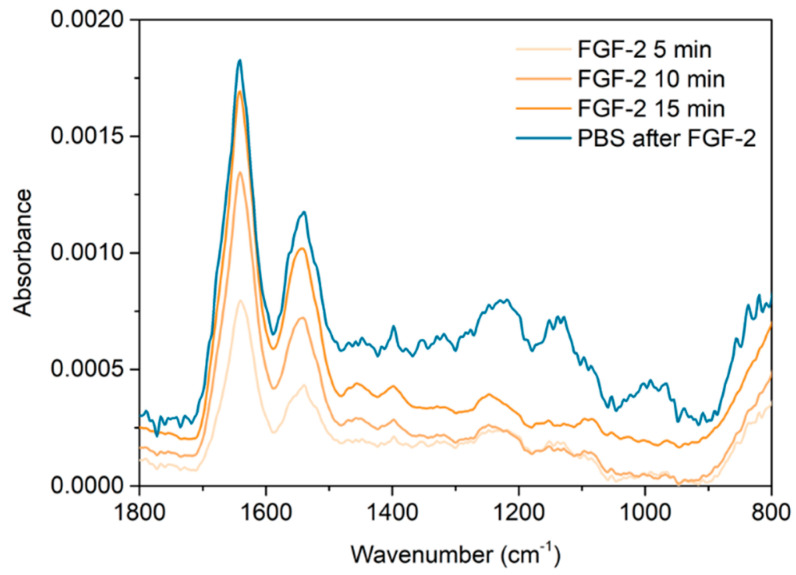
ATR FTIR individual layer spectra of the FGF-2 adsorption every 5 min in orange and the subsequent PBS rinse in blue. FGF-2 spectra are produced by 1 to 1 subtraction of the prior PBS rinse spectrum from each spectrum collected over the adsorption time, followed by subtraction of a PBS spectra to remove the O-H bending mode contribution of water.

The spectra presented in Figure 4, show more clearly the spectral change associated with the adsorption step of the growth factor. The amide I/II bands characteristic of proteins, in this case FGF-2, are sharp and clear, and are found at peak maxima of 1642 cm^−1^ and 1541 cm^−1^, respectively. The amide I/II bands increase over the 15 min adsorption. It is clear that FGF-2 has adsorbed to the surface of the multilayer. The subsequent PBS rinse showed no further change in the polysaccharide peaks or the amide bands suggesting the FGF-2 remained bound onto the multilayer surface and no further mass loss of the polysaccharides occurred. The FGF-2 exposure spectra will also include some contribution from the bulk solution above the PEM as well as any FGF-2 adsorbed to the PEM.

**Figure 5:**
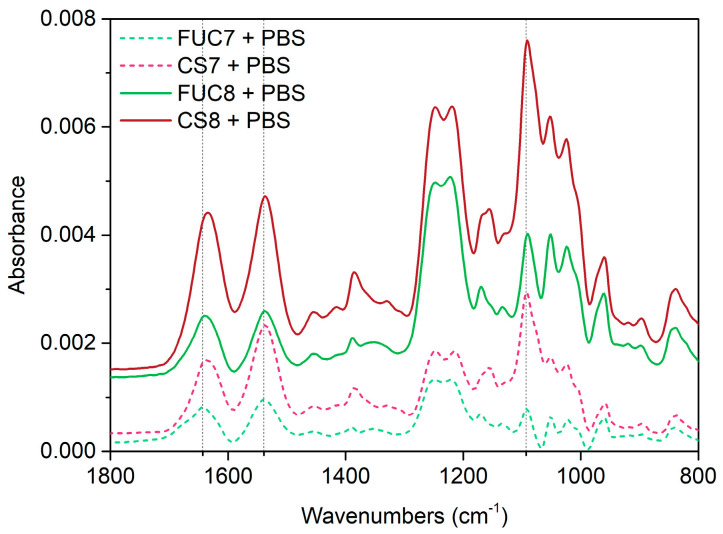
Representative ATR FTIR spectra of the layers added after FGF-2 adsorption. These spectra were produced by subtracting the spectra of the PBS rinse after FUC6 in a 1 to 1 ratio from each, followed by adding a PBS spectra to flatten the region between 1650–1700 cm^−1^ to remove the O–H bending mode of water lost during this adsorption step. Green lines show the fucoidan layers whilst red shows the chitosan layers. The vertical lines indicate the peak maxima of the amide I/II of FGF-2.

Finally, it was important to confirm that the FGF-2 remains bound within the PEM after build-up is continued, i.e., it is not removed by polyelectrolyte stripping. The spectra of the layers added after FGF-2 were processed by subtracting the spectra of the PBS rinse after FUC6 in a 1 to 1 ratio from each. These spectra are then processed to remove the O-H bending mode of water lost during this adsorption step, by summing the spectrum with a spectrum of PBS. Representative spectra are presented in Figure 5. Negative changes in the amide bands of the spectra in this figure may show if FGF-2 was desorbing or being removed from the multilayer.

The first FUC (seventh bilayer) adsorption after the embedded growth factor contains a clear sulfate band characteristic of fucoidan as would be expected, however, there is significant distortion in the region from 1100–900 cm^−1^ due to the overlapping nature of PBS peaks in this region. In addition, the amide I band peak maxima can be found at 1643 cm^−1^, whilst the amide II maxima is at 1539 cm^−1^, these peaks are indicative of FGF-2 remaining bound to the multilayer after fucoidan adsorption. Upon subsequent chitosan adsorption the amide I and II peaks became more rounded and the peak maxima of the amide I shifted to 1638 cm^−1^ but the amide II remained in the same position. Additionally, two peaks increase significantly at 1384 cm^−1^ and 1093 cm^−1^, assigned to the CH_3_ deformation and the C-O-C stretching mode of the glycosidic linkage overlapping with the symmetric stretching of phosphate in PBS (see electronic Supplementary Materials Figure S1). These peaks only increase with each chitosan addition. In the next fucoidan layer spectra the amide I band shifts back to 1640 cm^−1^, and both amide bands greatly reduce in size. This indicates that chitosan is removed from the film as was seen previously, while the shift towards the FGF-2-like amide I band suggests that the growth factor is still trapped within the film. The final adsorption of chitosan sees the amide I shift more dramatically to 1634 cm^−1^ due to significantly larger adsorbed amount.

These spectra also clearly show that the sharp peak at 1093 cm^−1^ is associated with chitosan adsorption. This sharp peak appears at the same wavenumber as the first of the glycosidic linkage peaks of the polyelectrolytes, i.e., the C-O-C and C-O stretching bands. However, this peak also overlaps with the symmetric stretching of phosphate in PBS [58], which is composed of two peaks at 1062 cm^−1^ and 1125 cm^−1^ (see electronic Supplementary Materials Figure S1).

In addition to determining that the FGF-2 does not release upon simple exposure to PBS, it is valuable to determine the likely structure and distribution of the FGF-2 within the multilayer. The presence of FGF-2 as a distinct layer within the multilayer will likely result in a different interaction within a wound environment compared to FGF-2 that is evenly distributed throughout the PEM film. In recently submitted work from our group, we determined that lysozyme was able to adsorb onto and permeate into a multilayer of FUC/CS (fucoidan/chitosan) [54]. Lysozyme and FGF-2 are both small proteins, with similar molecular weights and hydrodynamic radii; for lysozyme the molecular weight is 14.7 kDa and has a hydrodynamic radius of 19.5 Å, whilst FGF-2 has values of 17.2 kDa and 28 Å, respectively [9,28]. In addition, both have an overall positive charge at physiological pH, with isoelectric points at 11.3 for lysozyme and 9.6 for FGF-2. It was therefore our initial hypothesis that FGF-2 would behave similarly when a solution of the growth factor was placed in contact with a multilayer.

**Figure 6:**
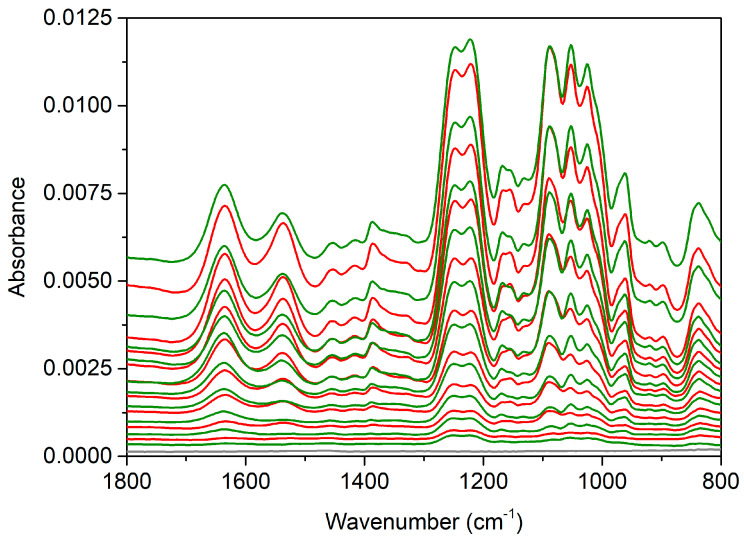
ATR FTIR spectra of the build-up of a 9.5 bilayer PEM on a Ge IRE. The spectra are produced by subtracting a spectrum of the background electrolyte from each spectrum collected after each polymer adsorption/rinse step. The spectrum of PEI is shown in grey, the spectra of the fucoidan layers are shown in green and the chitosan layers are shown in red.

**Figure 7:**
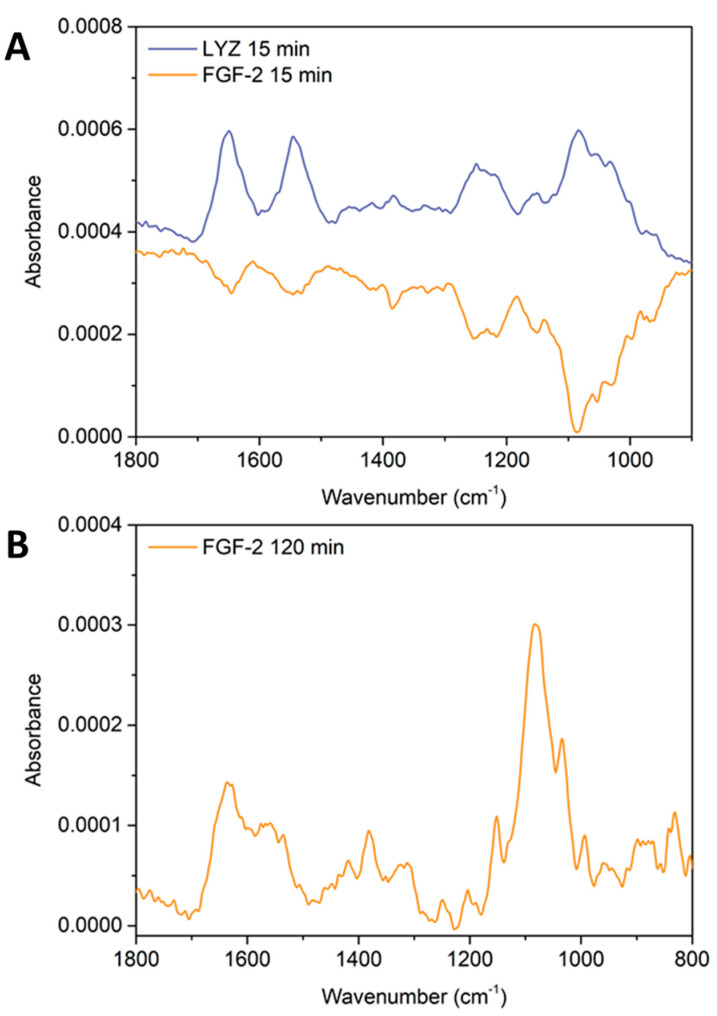
(**A**) ATR FTIR difference spectra of a 9.5 bilayer PEM exposed to PBS for 5 min followed by either lysozyme (dark blue) or FGF-2 (orange) for 15 min. (**B**) ATR FTIR difference spectrum of a 9.5 bilayer PEM exposed to PBS for 5 min followed by FGF-2 (orange) for 120 min. (Difference spectra acquired by subtracting the spectrum of the PBS exposed multilayer from the multilayer exposed to the two biomolecules).

To test this hypothesis, ATR FTIR spectroscopy on a Ge IRE was employed to monitor the build-up of a 9.5 bilayer FUC/CS PEM, similar to our previous experiments with lysozyme. The build-up was found to match the data from our earlier work, and is presented in Figure 6. The multilayer was then exposed to PBS solution for 5 min, and then a 25 μg·mL^−1^ FGF-2 solution (in PBS, which was used to maintain the secondary structure of the proteins) was injected into the flowcell and remained stagnant over the film for 15 min and a spectra collected of the bulk solution over the multilayer. The spectra of the protein exposure are presented in Figure 7 panel A, where a ratio of 1 to 1 was used to subtract the spectrum of PBS over FUC10 from the spectrum of 15 min protein exposure. The film was exposed to FGF-2 was exposed to PBS for 2 h and this spectrum is presented in Figure 7 panel B.

Upon fucoidan adsorption the sulfate peaks increase up to bilayer 4. From bilayer 5 onwards, the sulfate peak increases upon both fucoidan and chitosan adsorption. The amide I/II bands characteristic of chitosan increase upon adsorption of chitosan, but decrease upon subsequent adsorption of fucoidan throughout build-up, however, this becomes more noticeable in the latter layers. This is attributed to mass loss of chitosan via stripping by fucoidan as it adsorbs and swelling of the film. Swelling can be seen in ATR FTIR spectra where the penetration depth of the IRE is not much greater than the thickness of the film. This can also account for the increase in the sulfate peak after chitosan adsorption mentioned above.

The spectra in Figure 7 show that lysozyme can be detected within the multilayer, with amide I and amide II bands clearly visible at 1651 cm^−1^ and 1547 cm^−1^. In addition, the glycosidic linkage region of the polysaccharides can also be seen with a maxima at 1084 cm^−1^, as well as the sulfonate stretching band characteristic of FUC at 1215–1252 cm^−1^. In contrast, the FGF-2 spectrum shows negative bands at 1647 cm^−1^ and 1547 cm^−1^ assigned to the amide I/II of CS, as well as the negative sulfonate stretching band and the glycosidic linkage region with minima at 1215–1252 cm^−1^, and 1084 cm^−1^, respectively.

The refractive index of the Ge prism upon which these experiments were performed was *n*_Ge_ = 4.0, within the *ñ* = 700–3450 cm^−1^ range [59]. A high refractive index means that the penetration depth (*d*_p_) of the evanescent wave from the surface of the IRE in an aqueous environment is small, where *d*_p_ = 414 nm at *ñ* = 1540 cm^−1^ and *d*_p_ = 505 nm at *ñ* = 1250 cm^−1^ (see our previous work for all calculated *d*_p_ values and graphs of refractive indices for ZnSe and Ge IREs in the mid-IR range). In our earlier work, AFM measurements were used to determine the thickness of the 9.5 bilayer PEM in KCl electrolyte (377 ± 10 nm) and when exposed to PBS (432 ± 14 nm) [54]. These thickness measurements show that the multilayer is thicker than the penetration depth of the evanescent wave from a Ge IRE in the region of the amide I/II bands. Therefore, the spectra in Figure 7 shows that the film allows lysozyme to penetrate, as indicated by the amide I/II bands, and the PEM deswells as expected (and visualized by the increase in the polysaccharide peaks). However, the FGF-2 does not interact with the PEM in the same manner, the spectra indicates that the PEM is continuing to swell (likely due to the PBS) and is not counteracted by protein sorption (no positive amide I/II bands can be seen).

The FGF-2 bulk solution was allowed to remain on the film for 2 h (Figure 7 panel B), after this time the characteristic peaks of chitosan could be seen at 1636 cm^−1^ and 1558 cm^−1^, plus the glycosidic linkage region centred around 1080 cm^−1^. However, there appeared to be no amide I/II bands that matched the shape/ratios of the protein or any sulfate stretching bands indicative of fucoidan. This indicates that FGF-2 did not permeate into the film over this time frame and the film de-swelled into the evanescent wave. Specifically, a region/layer with high chitosan content, or that chitosan is diffusing through the PEM into the lower layers closer to the IRE surface. This diffusion may be facilitated by the long exposure to PBS meaning that the film remains in the swollen state during this time. Therefore, the chitosan may be freer to diffuse due to the higher degree of extrinsic versus intrinsic charge compensation and the ‘looser’ structure of the swollen PEM.

Since the our previous work determined that the PEM was able to exclude proteins based on size [54], the spectra presented in Figure 7 indicate that FGF-2 must interact via a different mechanism than lysozyme (LYZ) with the multilayer components since the difference in molecular weight and hydrodynamic radii between LYZ and FGF-2 is small. It must be also noted that heparin-like glycosaminoglycans (GAGs) (i.e., fucoidan) support dimerisation of FGF-2 which contributes to the potency of the growth factor in vivo [60]. However, our experiments could not distinguish between dimerised FGF-2 in contact with the fucoidan surface of the PEM, or whether monomers of FGF-2 were binding to the fucoidan on the films without dimerisation occurring.

## 3. Discussion

The data in this study clearly shows that FGF-2 does not permeate into the multilayer but can be embedded irreversibly at a desired location within the film. Both observations are likely due to the specific interactions between FGF-2 and heparin/or heparin-like mimics, i.e., fucoidan [33,61]. The FGF-2 protein has heparin-specific binding regions on the protein surface that contain lysine residues [62]. Under physiological conditions lysine residues are positively charged and can act as hydrogen bond donors [63]. The FGF family of growth factors all have different heparan sulfate glycosaminoglycan (HSGAG) binding domains [61]. The binding of FGF to HSGAGs is vital for binding to the tyrosine kinase receptors (FGFR) on cell surfaces as a ternary complex and regulating signalling [61]. FGF binding to an HSGAG oligosaccharide has been shown to involve both ionic and van der Waals forces, and was optimal due to the conformational changes of the HSGAG backbone that occur from protein binding, where the HSGAG kinks across 3 specific monosaccharide units, glucosamine-iduronate-glucosamine [61]. More recent work has shown that that binding of FGF to HSGAGs requires 3-*O*-sulfated glucosamine saccharide units, not specifically iduronate which has an additional carboxylate group in the 6-*O*-position [64]. *Fucus vesiculosus* fucoidan is predominantly comprised of long chains of 2-*O*-sulfated glucosamine units with some 3-*O*-sulfated glucosamines [50]. Thus, it is likely able to form the kinked structure around FGF proteins in a similar manner to the HSGAGs studied by Raman et al. [61]. The specificity of heparin binding domains in proteins such as FGF-2 result in quite different behaviour when compared to other proteins of similar size and charge that lack the specific heparin binding domains, i.e., lysozyme [65]. Another factor to consider is the oligermisation states of FGF-2, Kwan et al. reported that HSGAGs induced dimerisation of FGF-2 via surface-exposed cystine residues [60]. The HSGAGs stabilise the FGF-2 dimers and the dimers have a more potent effect than the monomeric form. If the FGF-2 is dimerising when in contact with fucoidan at the surface of the multilayer, this may contribute to the lack of diffusion into the lower layers of the film.

The work by Masuoka et al. showed that chitosan was able to protect FGF-2 from heat and enzymatic degradation at pH 7.3 (in PBS) but had no protective effect against acid degradation (pH < 5) [52]. This suggests that chitosan at or above its isoelectric point may be able to bind to FGF-2 as well. The amine groups of chitosan have an isoelectric point of 6.5 [66,67]. So, in PBS solution (pH 7.3) almost 50% of the amine groups will lose their positive charge. In our multilayers, if FGF-2 were binding to chitosan then when the pH is reduced to pH 5 upon return to the background electrolyte some loss of FGF-2 may be expected. However, this does not appear to be the case.

Other authors have seen similar results with other multilayer systems [68] where LYZ is able to permeate but FGF-2 does not, but, this is not always the case, and it is dependent on a multilayer structure [8,69,70,71]. Hsu et al. have published two works of interest, where either LYZ or FGF-2 were incorporated into the PEM structure as a component in a tetralayer [69]. In the first study, CS/poly(β-L-malic acid) (PMLA)/CS or LYZ/PMLA tetralayers were investigated with varying degrees of click crosslinking introduced via modified PMLA components to minimise interlayer diffusion and thus, control release of therapeutics [69]. Here, LYZ was used as a model protein, and was trapped in the lower part of the PEM by a cross-linked layer of PMLA. This barrier layer was able to suppress the burst release of protein and made the release duration longer, going from 2 to 3 days. Hsu’s second work, utilised the same PEM system with LYZ and FGF-2 [69]. In this study, they replaced LYZ with FGF-2 in the tetralayer PEM and found that more than six times less FGF-2 was incorporated than in similar LYZ films [69]. In addition, the loading of FGF-2 was linear with respect to film thickness. The release of FGF-2 had a similar profile to that of LYZ from the same films however, was of longer duration. The FGF-2 released from the films was found to have a greater proliferative activity than ‘as-received’ FGF-2, likely due to the co-release of chitosan, which may offer protective effects against heat denaturization [69].

Another group of authors who have created a body of work on the topic is Macdonald et al. who investigated PEMs comprised of a variety of synthetic and natural polyelectrolytes and their interactions with LYZ [70], FGF-2 [8] and BMP-2 [71]. In their 2008 paper, lysozyme was utilised as the polyanion in a tetralayer structure (polyX/polyanion/LYZ/polyanion)*_n_* where, *n* = 10–80 and polyX was one of two synthesised cationic poly(β-aminoesters), whilst the polyanion was, either heparin (HEP) or chondroitin [70]. The amount of LYZ incorporated was linear with film thickness. The same films were investigated with FGF-2 as the embedded protein in the tetralayer structure. It was found that poly2/HEP films contained the most FGF-2, partly due to the hydrophobicity of poly2 vs. poly1 which would result in less intrinsic charge compensation (poly2 films are thicker than poly1 due to this). In addition, films containing HEP can sequester more FGF-2 than the equivalent films containing chondroitin. It was proposed that the specific interactions between HEP and FGF-2 may be the dominant factor, however, in their previous work the HEP films were also able to load more LYZ than the chondroitin films [70]. So, the specific interactions between FGF-2 and HEP may not be the only reason why HEP multilayers were able to load more FGF-2 than the chondroitin films.

These studies correlate with our data confirming that heparin-specific binding sites play an important role in the uptake of FGF-2 to fucoidan/chitosan multilayer films and that fucoidan is likely acting as a heparin-mimic even when it is within a multilayer structure.

This is further confirmed by the FUC7 + PBS spectra in Figure 5, which shows that FGF-2 remains bound within the multilayer upon fucoidan adsorption, however, it is less clear in the subsequent spectra due to the overlapping amide bands of chitosan and FGF-2. Yet, it is still possible to assume the FGF-2 remains bound since upon addition of the seventh chitosan layer the amide I band does not shift as low as previously seen in earlier studies from our group [54] (to 1630 cm^−1^) and when the eighth fucoidan layer is adsorbed (and some chitosan is stripped) there is a shift in the amide I to higher wavenumbers i.e., closer to the peak maximum of FGF-2.

The sharp peak (Figure 5) assigned to the symmetric stretching of phosphate in PBS [58] (see electronic Supplementary Materials Figure S1) could suggest that some phosphate remains bound via ionic interactions to chitosan after the electrolyte is changed back to KCl. Laucirica et al. have shown that amine-phosphate interactions are specific and that this binding becomes apparent under physiologically relevant conditions [72]. In addition, the same work showed that the divalent HPO_4_^2−^ has an affinity for amino-groups that is five times greater than the monovalent H_2_PO_4_^1−^ ion due to the hydrogen bonding between the protons on the amine and the charged oxygen species of phosphate ions [72]. Peng et al. found similar results with molecular dynamics that showed that phosphate ions adsorb on to amino-terminated self-assembled monolayers but chloride ions do not [73].

## 4. Materials and Methods

### 4.1. Materials

Protosan UP CL 213, a chitosan salt that dissolves in water (CS, 75–90% deacetylated, 150–400 kDa) was sourced from NovaMatrix (Sandvika, Norway). Pharmaceutical grade *Fucus vesiculosus* fucoidan (FUC, Batch no. DPFVF2015505 from the Maritech ^®®^ range, 98% purity, 1.4% uronic acid, 56.9 kDa, 26.6% sulfate 50.7% fucose) was supplied by Marinova Pty Ltd. (Cambridge, TAS, Australia). The purification of fucoidan to remove pyrogens produces a highly pure material that is acceptable for medical use. Human fibroblast growth factor-basic (FGF-2, 154 a.a.) was supplied by Peprotech-Lonza, Mt Waverley, VIC, Australia. Lysozyme from chicken egg white (LYZ, dialyzed, lyophilized, powder, 100000 U∙mg^−1^) and polyethylenimine (PEI, branched, 25 kDa) and were obtained from Sigma-Aldrich, Australia.

Potassium chloride (KCl, 99%, AR) was purchased from Chem-Supply (Gillman, SA, Australia). The KCl was further purified to remove surface active impurities, by calcination at 550 °C for 8 h, followed by recrystalisation and finally, another calcination. Phosphate buffered saline (Dulbecco A) was obtained from Thermo Fisher Scientific, Adelaide, SA, Australia and used as supplied. HCl and KOH (both volumetric grade) were sourced from Merck KGaA, Darmstadt, Germany. Reagents used for cleaning surfaces include; ethanol 100% undenatured (AR, Chem-Supply, Gillman, SA, Australia), Hellmanex (Hellma Analytics, Müllheim, Germany), pH 7 Tickopur R 30 and OP-U colloidal silica suspension (Struers, Ballerup, Denmark).

### 4.2. Solution Preparation

Milli-Q water (resistivity: 18.2 MΩ∙cm; interfacial tension: 72.4 mN∙m^−1^ at 22 °C; total organic carbon content: < 4 mg∙L^−1^) was used to prepare all solutions and for cleaning of surfaces and glassware. The background electrolyte for the polyelectrolyte solutions was pH 5 0.1 M KCl solution (pH adjusted prior to making other solutions). PEI (500 ppm) was prepared in background electrolyte, stirred overnight and used within one week. Solutions of CS and FUC (both 500 ppm) were prepared in background electrolyte and stirred overnight. The polysaccharide solutions were used within 24 h of preparation. The background electrolyte, FUC and CS solutions were pH adjusted with volumetric grade KOH and HCl solutions to pH 5 before experiments. All pH adjustments were performed to give a value of ± 0.05 from the desired pH. PEI was used at its native pH in 0.1 M KCl pH 5 solution. Both LYZ and FGF-2 (both 25 ppm) were prepared the day of the experiment in PBS (pH 7.3) and stirred briefly to dissolve the protein.The concentration of 25 µg∙mL^−1^ FGF-2 solution was chosen for two main reasons; (i) physiological concentrations are approximately 50 pg∙mL^−1^ in plasma [74], (ii) spectroscopic detection levels were found to be in the range of hundreds of µg∙mL^−1^ for solution spectra. Typically, adsorption to a multilayer increases the concentration within the evanescent wave, and thus detection of less than this concentration is possible, it was decided to work in a range that would ensure detection. In addition other authors have found concentrations between 1.65–100 µg∙mL^−1^ growth factor solutions sufficient for multilayer studies [8,18,75,76,77].

### 4.3. Polyelectrolyte Multilayer Preparation and Growth Factor Adsorption/Incorporation

Multilayers were prepared, in situ, under flow for all experiments. Initially, the system is flushed with KCl background electrolyte, then an anchoring layer of PEI is deposited by flowing the solution over the substrate for 15 min followed by a 5 min rinse with KCl. Following the PEI layer, FUC is adsorbed then CS. Each polymer is adsorbed for 15 min followed by a 5 min KCl rinse. The fucoidan and chitosan layers make one bilayer pair. This bilayer is repeated until the desired bilayer number is reached. Where FGF-2 was embedded in the film, a total of 8 bilayers were used with a FGF-2 layer at bilayer 6 i.e., PEI-(FUC/CS)_5_-(FUC/FGF-2)-(FUC/CS)_2_. For the permeation experiments multilayers composed of 9.5 bilayers were used so the film can be described by; PEI-(FUC/CS)_9_-FUC.

### 4.4. Attenuated Total Reflectance Fourier Transform Infrared Spectroscopy (ATR FTIR)

Fourier transform infrared experiments were performed on a Varian 670-IR FTIR spectrometer (Agilent Technologies, Mulgrave, VIC, Australia). A Ge internal reflection element (IRE) was used for FGF-2 permeation experiments. A ZnSe IRE was used for the data shown throughout the main manuscript. The IRE was mounted in a Fast IR single reflection ATR accessory (Harrick Scientific, Pleasantville, NY, USA) and fitted with a liquid flow cell attached to a peristaltic pump (Masterflex L/S, John Morris Scientific, Deepdene, VIC, Australia) with Tygon tubing (Masterflex L/S 13, Cole Parmer, Vernon Hills, IL, USA).

The ZnSe/Ge IRE (Harrick Scientific, Pleasantville, NY, USA) was buffed in a figure-of-eight pattern for approximately 5 min with OP-U colloidal silica suspension on a wet, MD-Nap ™ 250 mm polishing pad (both Struers, Ballerup, Denmark), followed by buffing for a further 2 min with Milli-Q water. Each component was sonicated in a surfactant for 30 min; the IRE in 2% pH 7 Tickopur, the flow cell and the tubing were sonicated in 2% Hellmanex - each solution was injected through the tubing 3 times with syringes (Luer slip, 5 cc∙mL^−1^, Terumo, Tokyo, Japan). Each component was rinsed with Milli-Q water and then sonicated in 100% undenatured ethanol for 15 min (tubing was not exposed to ethanol), followed by a further rinse, then sonicated in Milli-Q water for 15 min. Finally, the components were rinsed a last time, dried under a stream of high purity dried nitrogen gas (99.999%, BOC, North Ryde, NSW, Australia) and allowed to dry fully overnight in a covered plastic container before being mounted.

Multilayers were created on the IRE surface under flow by following the protocol outlined above. The FGF-2 adsorption and PBS rinses were performed by flowing PBS over the multilayer for 5 min at 1.000 mL∙min^−1^. Then the tubing was removed and FGF-2 solution was injected directly into the flowcell chamber via a syringe (Luer slip, 1 cc∙mL^−1^, Terumo, Japan). The injection of the solution was staged over the 15 min adsorption, with 0.3 mL injected at 0, 5 and 10 min. For the diffusion study the FGF-2 remained on the multilayer for 2 h. The tubing was reconnected to the flowcell and then flushed with PBS again for 5 min at 1.000 mL∙min^−1^.

Single channel spectra from 256 scans were obtained in the region of 650 cm^−1^ (on the ZnSe IRE) or 780 cm^−1^ (on the Ge IRE) to 4000 cm^−1^, with 4 cm^−1^ resolution (commonly employed for studies of condensed matter systems, as higher resolution does not provide finer detail of peaks due to the natural linewidth of peaks in such systems) using Agilent Resolutions Pro software v5.2.0.36. Spectra were recorded for each experiment, as follows; (i) a background spectrum in air; (ii) a water vapour (WV) spectrum in air 10 min after the background spectrum; (iii) a spectrum of the background electrolyte after 5 min flow; (iv) then polymer spectra after each successive adsorption/rinse cycle.

Spectra were collected at specific time points, during the PBS/FGF-2/PBS cycle; (i) after the 5 min PBS rinse of the 5.5 bilayer, fucoidan terminating PEM, (ii) every 5 min during FGF-2 adsorption and (iii) after the 5 min PBS rinse after growth factor adsorption. These spectra were processed by subtracting the initial PBS rinse from the FGF-2 and subsequent PBS rinse with a 1 to 1 ratio, then a spectrum of PBS was added to flatten the O-H bending mode of water.

Whilst for the diffusion study on the 9.5 bilayer multilayer, spectra were collected at 15 min FGF-2 adsorption and then after a final 5 min PBS rinse following the 2 h FGF-2 adsorption. These spectra were processed by subtracting the PBS rinse of the multilayer in a 1 to 1 ratio from all subsequent spectra. Each experiment was performed as two independent repeats. Spectral processing was performed with OMNIC software v8.2.0.387 (Thermo Fisher Scientific, Scoresby, VIC, Australia).

The multilayer build-up spectra presented below (Figure 3) and in the electronic supplementary material were produced by subtracting the spectrum of the background electrolyte (KCl) from each spectrum to remove the contribution of water in the O-H bending mode region (~1630 cm^−1^). In Figure 4 and Figure 5 the spectra were produced by subtracting the spectra of the PBS rinse of the 6th fucoidan layer (PBS rinse after FUC6) in a 1 to 1 ratio from each, followed by adding a PBS spectra to flatten the region between 1650–1700 cm^−1^ to remove the O-H bending mode of water. Finally, manual water vapour correction was performed, followed by automatic baseline correction, for all spectra.

### 4.5. Quartz Crystal Microbalance with Dissipation Monitoring (QCM-D)

The experiments were performed on a Q-sense E4 instrument (Biolin Scientific, Västra Frölunda, Sweden) under continuous flow conditions. The multilayers are formed on Si-coated 5 MHz AT-cut quartz crystal sensors (SiO_2_ 50 nm, QSX 303, Q-sense, Biolin Scientific, Sweden). The sensors were cleaned by sonicating in 1 M HCl for 30 min, followed by 2% Hellmanex for 30 min then Milli-Q water for 10 min. The sensors were individually dried under a stream of high purity dried nitrogen and air plasma cleaned for 60 s (Harrick Plasma, Ithaca, NY, USA).

Once cleaned, the sensors were placed into the QCM chambers, where they were allowed to stabilise in background electrolyte prior to measurement for 1 h under flow at 0.050 mL∙min^−1^ using a multi-channel peristaltic pump (Ismatec, Cole-Palmer, Wertheim, Germany). Then solutions were pumped through the system following the adsorption protocol described above at rates of 0.100 mL∙min^-1^ for polyelectrolyte/protein adsorption and 0.300 mL∙min^−1^ for the background electrolyte rinse.

The PBS/FGF-2/PBS cycle was performed using a flow rate of 0.300 mL∙min^−1^ for 5 min for the PBS rinse prior to the adsorption of growth factor. The FGF-2 adsorption was performed by flowing the growth factor solution over the multilayer for 1 min at a rate of 0.30 mL∙min^−1^, then for 14 min at a rate of 0.05 mL∙min^−1^. The subsequent PBS flush was performed at 0.05 mL∙min^−1^ and 0.30 mL∙min^−1^ for 5 min each (the extra slow flush was used to account for the additional exposure time of FGF-2 due to spectra collection in ATR FTIR spectroscopy measurements).

## 5. Conclusions

The ATR infrared spectra and quartz crystal microbalance measurements presented show that fibroblast growth factor-2 can be embedded into the film structure by adsorbing layers of polyelectrolytes over the FGF-2. In addition, it was found that the overall multilayer structure is altered by PBS exposure during the embedding process, and that the incorporation of growth factor had little disruptive effect on the film build-up. This ability of fucoidan to bind fibroblast growth factor 2 in a multilayer structure could offer new methods for protecting and deploying growth factor in a wound bed. Such deployment as a coating on wound dressing material would allow the growth factor to be active in a wound bed as the polyelectrolyte degrades and exposes/releases the growth factor.

## Figures and Tables

**Table 1:** Assignment of bands observed for ATR FTIR (attenuated total reflectance Fourier transform infrared) spectra of (i) a 9.5 bilayer fucoidan/chitosan polyelectrolyte multilayer on a Ge IRE (internal reflection element) and (ii) an 8 bilayer fucoidan/chitosan polyelectrolyte multilayer with FGF-2 embedded at bilayer 6 built on a ZnSe IRE [27,30,31,32,33,34,35,36,37,38,39,40]. Annotations: *ν* is stretching vibration, *ν*_as_ is asymmetric stretching vibration, *ν*_s_ is symmetric stretching vibration, *γ* is out-of-plane bending vibration, *δ* is in-plane bending vibration.

Peak Assignment	9.5 BL Chitosan/Fucoidan (Ge IRE)	8 BL Chitosan/Fucoidan with FGF-2 Embedded at BL 6 (ZnSe IRE)
*ν*_s_(C-O-S)	838	838
*ν*_s_(C-H)	898	898
*ν*(C-O), *ν*_s_(C-O-S)	961	961
*ν*(C-O-C), *ν*(C-O), *ν*(C-C)	1025	1024
*ν*(C-O-C), *ν*(C-O), *ν*(S = O)	1052	1052
	1089	1091
*ν*(C-N), *γ*(C-O-C)	1155	1155
*γ*(C-O-C)	1167	1167
*ν*_as_(S = O)	1222	1220
	1248	1248
*γ*(CH_3_)	1386	1386
*δ*(CH_2_)	1416	1416
*δ*(CH_2_)	1454	1454
Amide II, *δ*(N-H), *ν*_as_(COO^-^)	1538	1536
Amide I, *δ*(O-H)	1632	1635

## Supplementary Materials

The following are available online at https://www.mdpi.com/1660-3397/18/11/531/s1, Figure S1: ATR FTIR spectrum.
