# Supplementary material for: Incorporation of FGF-2 into Pharmaceutical Grade Fucoidan/Chitosan Polyelectrolyte Multilayers
Source: Mar Drugs. 2020 Oct 26;18(11):531. doi: 10.3390/md18110531 (PMC7692699; doi:10.3390/md18110531)
Supplement: Supplementary file 1 [file marinedrugs-18-00531-s001.pdf]

marine drugs

Supplementary Material

Incorporation of FGF-2 into Pharmaceutical Grade Fucoidan/Chitosan Polyelectrolyte Multilayers

Natalie L. Benbow [1], Samuel Karpiniec [2], Marta Krasowska [1,*] and David A. Beattie [1,*]

[1] Future Industries Institute, University of South Australia, Mawson Lakes, SA 5095, Australia
[2] Marinova, Cambridge, TAS 7170, Australia
* Correspondence: Marta.Krasowska@unisa.edu.au (M.K.); David.Beattie@unisa.edu.au (D.A.B.)

ATR FTIR

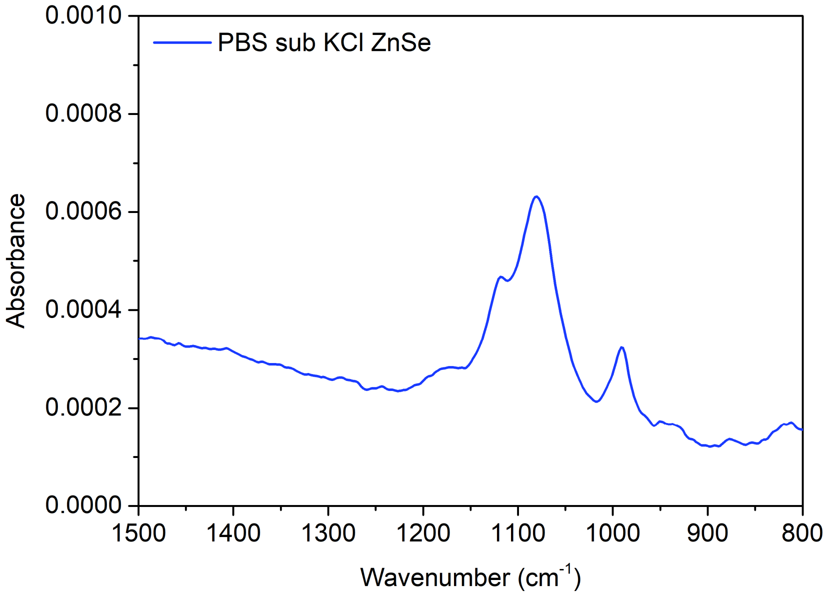

Figure S.1 ATR FTIR spectrum of PBS on a ZnSe IRE after a KCl spectrum has been subtracted to remove the contribution of the O-H bending mode of water. This spectrum has been corrected for water vapour contributions.

© 2020 by the authors. Licensee MDPI, Basel, Switzerland. This article is an open access article distributed under the terms and conditions of the Creative Commons Attribution (CC BY) license (http://creativecommons.org/licenses/by/4.0/).
